# A Bi-Exponential Repair Algorithm for Radiation-Induced Double-Strand Breaks: Application to Simulation of Chromosome Aberrations

**DOI:** 10.3390/genes10110936

**Published:** 2019-11-16

**Authors:** Ianik Plante, Tony Slaba, Zarana Shavers, Megumi Hada

**Affiliations:** 1 KBR, 2400 NASA Parkway, Houston, TX 77058, USA; zarana.s.patel@nasa.gov; 2 NASA Langley Research Center, Hampton, VA 23681, USA; tony.c.slaba@nasa.gov; 3 Radiation Institute for Science & Engineering, Prairie View A&M University, Prairie View, TX 77446, USA; mehada@PVAMU.EDU

**Keywords:** DNA double-strand breaks, ionizing radiation, repair kinetics, bi-exponential, chromosome aberrations

## Abstract

Background: Radiation induces DNA double-strand breaks (DSBs), and chromosome aberrations (CA) form during the DSBs repair process. Several methods have been used to model the repair kinetics of DSBs including the bi-exponential model, i.e., *N(t) = N_1_exp(−t/τ_1_) + N_2_exp(−t/τ_2_)*, where *N(t)* is the number of breaks at time *t*, and *N_1_*, *N_2_*, *τ_1_* and *τ_2_* are parameters. This bi-exponential fit for DSB decay suggests that some breaks are repaired rapidly and other, more complex breaks, take longer to repair. Methods: The bi-exponential repair kinetics model is implemented into a recent simulation code called RITCARD (Radiation Induced Tracks, Chromosome Aberrations, Repair, and Damage). RITCARD simulates the geometric configuration of human chromosomes, radiation-induced breaks, their repair, and the creation of various categories of CAs. The bi-exponential repair relies on a computational algorithm that is shown to be mathematically exact. To categorize breaks as complex or simple, a threshold for the local (voxel) dose was used. Results: The main findings are: i) the curves for the kinetics of restitution of DSBs are mostly independent of dose; ii) the fraction of unrepaired breaks increases with the linear energy transfer (LET) of the incident radiation; iii) the simulated dose–response curves for simple reciprocal chromosome exchanges that are linear-quadratic; iv) the alpha coefficient of the dose–response curve peaks at about 100 keV/µm.

## 1. Introduction

### 1.1. Importance of the Repair Kinetics Algorithm

Ionizing radiation creates various types of DNA breaks in cells, notably double-strand breaks (DSBs). These DSBs are repaired by different mechanisms: homologous recombination (HR) and non-homologous end joining (NHEJ) pathways, and others [1]. Indeed, DNA repair mechanisms have been intensely studied for many years [2,3,4]. Many theories have been proposed to explain the biphasic-like shape of repair curves observed experimentally (reviewed in [1]). These theories hypothesize that two types of DNA damage exists, each characterized by a single and constant repair half-life. It should be noted that transient DSBs can also be formed during the repair process by the endonucleases [5]; however, they are not considered in this work. One important consequence of non-homologous repair is improper rejoinings, which may lead to the loss of nucleotides and the formation of chromosome aberrations (CA).

We recently developed a code called RITCARD (Radiation Induced Tracks, Chromosome Aberrations, Repair, and Damage) [6] to simulate radiation-induced CAs. RITCARD uses a repair kinetics model that was carried over from the code NASA Radiation Track Image (NASARTI) [7,8]. Although this algorithm effectively simulates DSB repair, so that the number of DSBs decays as a function of time, that decay is not bi-exponential. The shape of the curve for the kinetics of restitution of DSBs given by NASARTI’s algorithm also depends on the radiation dose, and it rarely predicts unrepaired breaks 24 hours after irradiation, which is not in agreement with current experimental results [9,10,11]. Nevertheless, NASARTI and RITCARD calculations predict realistic yields of chromosome aberration for some types of monoenergetic radiation.

This paper describes how to simulate the repair kinetics of breaks with mixture of fast and slow repair, i.e., a bi-exponential decay, and how to apply this restitution kinetics algorithm to the simulation of chromosome aberrations. The algorithm presented here can generate the bi-exponential decay curve exactly and could be easily generalized to include three or more exponential repair kinetics. Furthermore, since the repair probabilities are not a function of the time at repair, but only the time interval, the algorithm could potentially be used in dose-rate studies. The calculations of radiation-induced CA were then performed using the code RITCARD, with the new repair kinetics algorithm. Dose–response curves were obtained for simple exchanges for 250 MeV protons, 250 MeV/n helium, 350 MeV/n oxygen and 300 MeV/n titanium ions, and were compared with the results obtained with the previous algorithm and experimental data. More dose–response calculations were performed for the ions H, He, C, N, O, Si, Ti, and Fe, at energies varying from 1 to 1000 MeV/n, covering a wide range of LET values. The dose–response curves were modeled by a linear-quadratic model. The linear coefficient of the dose–response curve peaks at 100 keV/µm. 

### 1.2. The Code RITCARD

NASA’s efforts to simulate and model radiation-induced CAs spans several years [6,7,8]. The recently developed RITCARD code is largely based on the previously developed NASARTI code. The main application of the new bi-exponential repair algorithm is for use in the code RITCARD, which is described briefly here; the details are given in [6]. This code is composed of three main parts. 

The RITCARD model first simulates the chromosomes using a random walk (RW) algorithm, identical to the process used in NASARTI, and then simulates voxel dose in the irradiated volume using the RITRACKS (Relativistic Ion Tracks) code. A discrete RW process is used to simulate the chromosomes, meaning that each chromosome is approximated by a coil comprising a series of monomers of 0.02 µm, each monomer containing 1000 base pairs of DNA. The nucleus can be modeled either as a sphere (e.g., for lymphocytes) or as an ellipsoid (e.g., for fibroblasts). The RITRACKS code simulates the radiation tracks as described in reference [12] and references therein. RITRACKS also calculates the dose in voxels of 20 nm, which matches the size of the monomer units of the chromosomes simulated by RW. Assuming that radiation-induced breaks occur exclusively at the intersections between the tracks and the chromosomes, the numbers of DSBs present in a monomer located in a voxel is given by the Poisson distribution (1):

(1) $$P=\frac{\lambda^{n}}{n!}e^{-\lambda },$$

where *λ = Q⋅D(i,j,k)*, *D(i,j,k)* is the local (voxel) dose, *i*, *j*, and *k* are the spatial coordinates of the voxel (in lattice units), and *Q* is an adjustable parameter representative of the intensity of the stochastic process of DSB formation, so that the number of DSBs is ≈35 DSBs/Gy/Cell. In this work, *Q = 1.14 × 10^−5^ Gy^−1^* and does not change with LET. This model of chromosome–voxel intersection breaks is illustrated in [13]. Each break leads to the formation of two new free ends in the broken chromosome. The number of breaks in a monomer is small and rarely greater than 1, except in the case of high-LET radiation. In the previous versions of RITCARD and NASARTI, the conditions required for a simulation of a chromosome break were slightly different and calculated as at least one DSB in the monomer.

The RITCARD code then simulates the rejoining process. Any of the free ends in the system can join (repair), and the restitution kinetics model imposes the probability of joining. The algorithm used in NASARTI and in the first version of RITCARD are briefly described below.

The NASARTI program and the previous version of RITCARD used time steps of 5.18 s, for 24 hours. This time step was determined from prior calibration of the model, during which the DSB repair kinetics were fitted as a bimodal exponential curve [7]. At each time step, the program randomly picks two free ends from those present in the system at that time. If the free ends originate from the same break, the rejoining is considered proper repair (i.e., the original break ends are rejoined) and is repaired with probability *P = 1*. Otherwise, the rejoining is considered improper repair. The probability of improper repair depends on the three-dimensional (3D) Euclidian distance between broken ends as determined using Equation 2:

(2) $$I=\frac{1}{W}e^{-r^{2}/\sigma^{2}},$$

where *I*, is the probability of an improper rejoining (misrejoining) in an elementary step of the algorithm; *W* is an empirically calibrated parameter; *r* is a 3D Euclidian distance between reacting DSB free ends (µm); and *σ* is an adjustable parameter with the dimension of microns. For a break to repair, both corresponding free ends must have been created at the time of repair. This condition is added to account for dose-rate effects. When a pair of free ends is repaired, all free end pairs containing these free ends are removed from the list of free end pairs in the system, because one of the free ends constituting the free end pair is already repaired and, therefore, cannot participate in the repair process any further. 

Lastly, the RITCARD code regroups the fragment sequences generated by the repairs (at 24 hours), analyzes them to find CAs, and counts the different types of CAs. Since this topic is beyond the scope of this article, it will not be discussed further here. The chromosome classification used in RITCARD essentially follows the one given in reference [14]. An exchange was defined as simple if it appeared to involve two breaks in two chromosomes, i.e., dicentrics and translocations. Incomplete translocations and incomplete dicentrics were included in the category of simple exchanges, assuming that in most cases, the reciprocal fragments were below the level of detection [15].

## 2. Materials and Methods 

### 2.1. The Bi-Exponential Decay Model 

The time-course of repair of radiation-induced DSBs in mammalian cells have been described by many investigators as being biphasic [1]. Therefore, for this discussion, it is assumed that two types of breaks (type 1, or fast repair of mean lifetime *τ_1_* and type 2, or slow repair of mean lifetime *τ_2_*) are present in the system. To introduce the algorithm, we assume that they are all independent (i.e., the free ends that are repaired need to originate from the same initial break). The latter restriction was lifted later to allow the simulation of improper repairs. For further simplification, acute irradiation scenario is used, so that all chromosome breaks are created at the time *t = 0*. Hence, in a simple exponential decay model, the number of breaks of type 1 that survive up to time *t*, denoted *N_1_(t)*, is given by

(3) N1(t)=N1(0)exp(−tτ1).

Similarly, the number of breaks of type 2 that survive up to time *t*, denoted *N_2_(t)*, is given by

(4) N2(t)=N2(0)exp(−tτ2).

The total number of breaks remaining in the system at time *t* in a bi-exponential decay model is, therefore,

(5) N(t)=N1(t)+N2(t)=N1(0)exp(−tτ1)+N2(0)exp(−tτ2).

Here $N_{1}\left(0\right)/N\left(0\right)$ and $N_{2}\left(t\right)/N\left(0\right)$ are the initial fraction of breaks of types 1 and 2. 

Since $S_{1}\left(t\right)=N_{1}\left(t\right)/N_{1}\left(0\right)=\exp\left(-t/\tau_{1}\right)$ is the survival probability for type 1 breaks, then the cumulative probability of repair at time *t* is

(6) F1(t)=1−S1(t)=1−exp(−tτ1).

The hazard function, h(t), is the instantaneous rate at which events occur, given that there were no previous events. In the context of this program, an event here means that a break is repaired. Similarly, the cumulative hazard H(t) describes the accumulated risk (of repair) up to time *t*. For type 1 breaks, these quantities can be calculated as in Equation (7):

(7a) H1(t)=−log(S1(t))=tτ1.

(7b) h1(t)=−∂∂tlog(S1(t))=1τ1.

Similarly, for type 2 breaks, $H_{2}\left(t\right)=t/\tau_{2}$ and $h_{2}\left(t\right)=1/\tau_{2}$.

### 2.2. The Monte-Carlo Simulation Algorithms for the Repair of Breaks

To generate the repair kinetics for a mixture of types 1 and 2 breaks, as given by Equation (5), simply generating *N_1_(0)* and *N_2_(0)* breaks of type 1 and 2 and adding their contributions directly does not work. Instead, their fractions have to be weighted by their contribution to the integral curve. More details are given in Appendix A. Specifically,

(8a) A1=∫0∞N1(0)exp(−tτ1)dt=N1(0)τ1.

(8b) A2=∫0∞N2(0)exp(−tτ2)dt=N2(0)τ2.

(8c) $$A=A_{1}+A_{2}=N_{1}\left(0\right)\tau_{1}+N_{2}\left(0\right)\tau_{2}$$

Therefore, the simulation of the repair kinetics must be performed as described in Algorithm 1.

**Figure 1:**
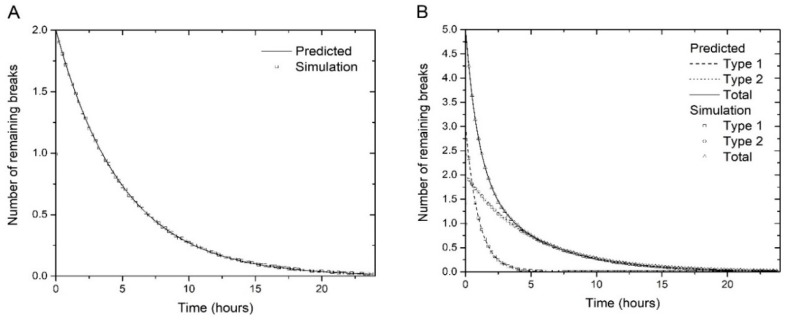
(**A**) Simulation of repair kinetics of breaks using Algorithm 1, with parameters *N_1_(0) = 2*, *N_2_(0) = 0* and *τ_1_ = 5 h* (i.e. one type of breaks) (**B**) Repair kinetics of type 1 and 2 breaks, and the total number of breaks, as simulated with algorithm 1, with parameters *N_1_(0) = 3*, *τ_1_ = 1 h*, *N_2_(0) = 2*, *τ_2_ = 5 h*. The number of simulation histories in both cases is 1,000,000. All simulation curves decay as predicted by the corresponding analytical curves (Equations (3)–(5)).

In Algorithm 1, the repair probabilities are determined by $\Delta t/\tau_{1}$ and $\Delta t/\tau_{2}$. We tested the algorithm with several values of the parameters. Figure 1 A and B show examples performed with 1,000,000 histories for one and two types of breaks, and illustrates the convergence of the simulation results to the corresponding predicted curves. This algorithm could be easily extended to include additional exponential decay contributions.

**Algorithm 1**: Simulation of repair kinetics for a mixture of two types of breaksSET *t_final_*, *Δt* << *t_final_*SET *A_1_* = *N_1_(0)τ_1_*, *A_2_* = *N_2_(0)τ_2_*, and *A* = *A_1_* + *A_2_*REPEAT *N_histories_* times {  GENERATE one break  GENERATE a uniform random number *R_1_* between 0 and 1  IF (*R_1_* < *A_1_/A*) {    SET type = 1    SET *p_Repair_ =*
*Δt/τ_1_* (essentially, $h_{1}\left(t\right)\Delta t$)  } ELSE {    SET type = 2    SET *p_Repair_* = *Δt/τ_2_* (essentially, $h_{2}\left(t\right)\Delta t$)  }   *t* = *0*
  *Repaired = false*;  REPEAT UNTIL *Repaired = true* OR *t > t_final_* {    GENERATE a uniform random number *R_2_* between 0 and 1    If *R_2_* < *pRepair* {     *Repaired = true*;      RECORD *t_repair_ = t* (the break is repaired) for type 1 or 2    }    *t* = *t* + *Δt*  }}GENERATE histogram of repaired times (total, type 1 and type 2)

It should be noted that the linear congruential random number generator does not work well for Algorithm 1, as some correlations exists between the pseudo-random numbers generated. After testing, it is recommended to use the Mersenne Twister (which is now a standard function in C++) for generating random numbers for this algorithm.

### 2.3. Application to Simulation of Chromosome Aberrations

The second part of the RITCARD program is the simulation of break repair. This was done previously using the simulation algorithm described in references [7,8]. A rigorous analysis of this algorithm, however, revealed that the repair of breaks does not follow a bi-exponential curve. Furthermore, the shape of the curve given by this algorithm depends on the initial number of breaks. Therefore, a new fit with different parameters was needed for each radiation dose. Another notable problem is that all breaks are usually repaired within 24 hours of irradiation, which is not in agreement with actual experimental results.

**Figure 2:**
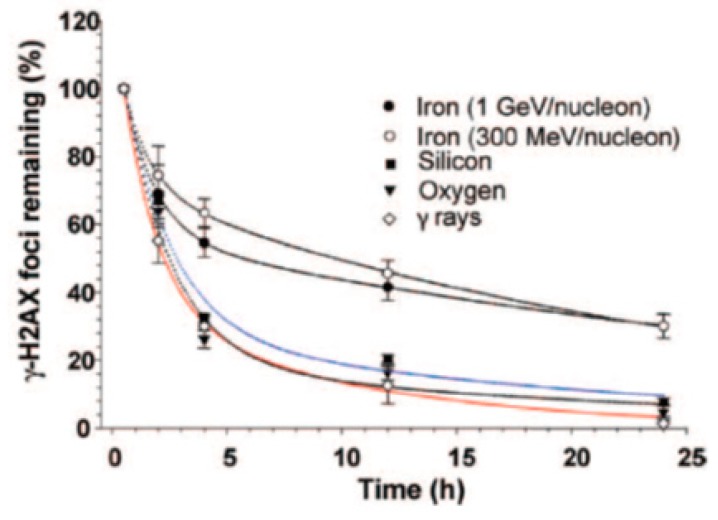
Quantification of γ-H2AX foci in human skin fibroblasts exposed to 1 Gy of iron, silicon, oxygen particles and γ-rays. Reproduced with permission^©^ 2019 Radiation Research Society.

We used published repair kinetics data [9], shown in Figure 2, to determine the parameters for the new algorithm, as explained below.

The values of the parameters for the bi-exponentials decay functions ($N=a_{1}e^{-t/\tau_{1}}+a_{2}e^{-t/\tau_{2}}$) used in reference [9] are given in Table 1.

Assuming that type 1 breaks are simple to repair (small *τ_1_*), and type 2 breaks are more complex to repair (large *τ_2_*), the following approach was used to implement algorithm 1 into the code.

**Figure 3:**
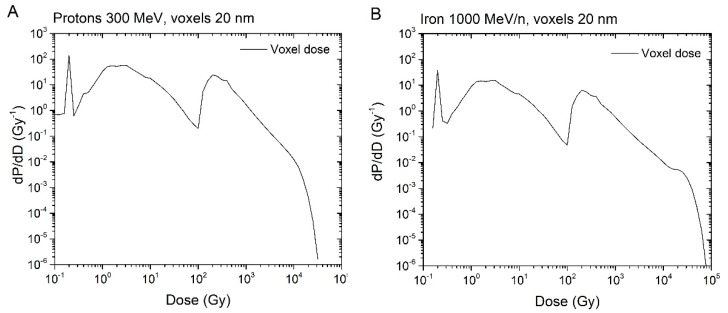
Dose distribution (dose per voxel) for 300 MeV protons (**A**) and 1000 MeV/n iron ions (**B**)

**Figure 4:**
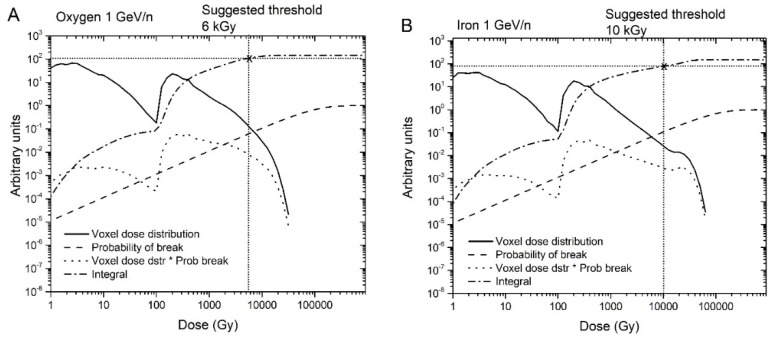
Dose distribution per voxel, probability of at least one break, voxel dose distribution x probability of at least one break, and cumulative integral of the previous quantity for oxygen 1 GeV/n (**A**) and iron 1 GeV/n (**B**).

- The repair times *τ_1_* and *τ_2_* are intrinsic for the repair mechanisms for a given type of cell. Therefore, we take the average from several curves for *τ_1_* and *τ_2_*. For the data given in Table 1, the averages for *τ_1_* and *τ_2_* are 1.7 hours and 23.7 hours, respectively.
- The data suggest that simple breaks are of type 1, and complex breaks are of type 2. The notion of “clean” and “dirty” breaks, used in reference [16], is a similar concept. The question was to determine which breaks are type 1 and which are type 2. We hypothesize that a higher voxel dose is needed to create a complex break. The dose in each individual voxel is itself dependent on different factors, and high-dose voxels are usually those located in the core of high LET tracks.
- To classify a voxel dose as high or low, we first need to determine a cut-off dose. To do this, we referred to the dose distribution per voxel (i.e., the voxel dose histogram). As an example, the energy distribution in voxels for 300 MeV protons and 1000 MeV/n irons calculated by the code RITRACKS are illustrated in Figure 3. It is worth mentioning here that other researchers have calculated the same shape of dose distribution per voxel, e.g., reference [17].
- The probability of creating *n* breaks is given by Equation (1), which implies that the probability of creating at least one break is *1-exp (-λ)*. Hence, the expected energy distribution in voxels corresponding to chromosome breaks can be estimated by weighting the probability of creating at least one break in a voxel by the voxel dose distribution.
- Using the dose distribution per voxel and the probability of creating at least one break, we can establish an energy threshold for each type of radiation that would determine the break type. Specifically, if the energy in a voxel is over the threshold, the break is type 2; otherwise, it is type 1. As shown in Figure 4, we accomplished this using the cumulative (integrated) curve of the energy distribution in breaks. For Iron 1 GeV/n, a threshold of around 10 kGy would result in a fraction of 0.52 breaks of type 1. We repeated this process for the other radiations in Table 1, and determined threshold values that varied from 6 kGy to 10 kGy. Although 6 to 10 kGy may seem a large range, the graphs are plotted on a logarithmic scale, and the threshold values are only 2–3 bins away from each other. We used a threshold dose of 10 kGy corresponding to a voxel energy deposition of 500 eV for the calculations reported in the present manuscript.
- In a subsequent step, we assume that breaks that repair fast result in proper rejoinings. Therefore, all the breaks belonging to this category are considered independent of each other. Improper rejoinings involve different repair enzymes or processes and are considered complex type repairs in our model. Therefore, the following modifications to the repair algorithm in RITCARD were implemented:
  - The fast repaired breaks are exclusively proper rejoinings.
  - Slow repaired breaks can be either proper or improper rejoinings. When free ends join via the slow process, a list of probabilities is assigned to other free ends originating from complex breaks. As described in references [7,8], the probability of rejoining is proportional to *exp(-distance^2^/σ^2^)*, so that closer free ends have a higher probability of recombination, the highest being the pairs originating from the same break (*distance = 0*);
  - Since improper repairs happen strictly in the pool of complex breaks, the overall repair kinetics remains the same (improper repairs were not permitted in the initial implementation of the algorithm, which was done for testing purposes);
  - Because each free end pair is counted twice while looping through the breaks at each time step, the overall probability of improper rejoinings is multiplied by 0.5.

This can be summarized in Algorithm 2.

**Algorithm 2**: Simulation of repair kinetics for breaks in RITCARD for one simulation historySET *t = 0*, *t_final_*, *Δt << t_final_*GENERATE all chromosome breaks and the list of corresponding (unrepaired) free ends.REPEAT for each free end {  DETERMINE whether the free end is simple or complex by using the dose threshold }REPEAT UNTIL *t = t_final_* {   SHUFFLE the free ends in the list.   SELECT the next unrepaired free end   IF simple {      Attempt repair with probability *p_Repair_ =*
*Δt/τ_1_*
     IF (repaired) {      REMOVE the free end and the corresponding free end from the list of unrepaired free ends     }  }  ELSE {      Attempt repair with probability *p_Repair_ = 0.5×Δt/τ_2_*
     IF (repaired) {       GENERATE a list of all other unrepaired complex free ends      Assign a probability of repair for each free end pair as *exp (-distance^2^/σ^2^)*, and normalize the probabilities so that the sum is equal to 1       GENERATE a random number to determine which free end pair is repaired      REMOVE the free end and the corresponding free end from the list of unrepaired free ends     }  }  *t = t + Δt*}

### 2.4. Additional Simulation Details

The simulations were performed as described in the original RITCARD paper [6]. Specifically, we simulated human fibroblast cells using a 17 µm × 17 µm × 6 µm box for the shape of the nuclei. We simulated exposures of H, He, C, N, O, Si, Ti, and Fe ions with energies varying from 1 MeV/n to 1000 MeV/n, covering LET values from 0.22 to 2363 keV/µm. To obtain a dose–response curve for each ion type and energy, we simulated 0.05, 0.1, 0.2, 0.3, 0.4, and 0.5 Gy doses. For each simulation condition, 10,000 histories are usually sufficient to ensure convergence of the CA yields for simple exchanges. The error bars for the simulated points were obtained as *1.96σ*, where *σ* is the variance of the simulated results.

## 3. Results and Discussion

### 3.1. Calculated DSB Decay Curves

**Figure 5:**
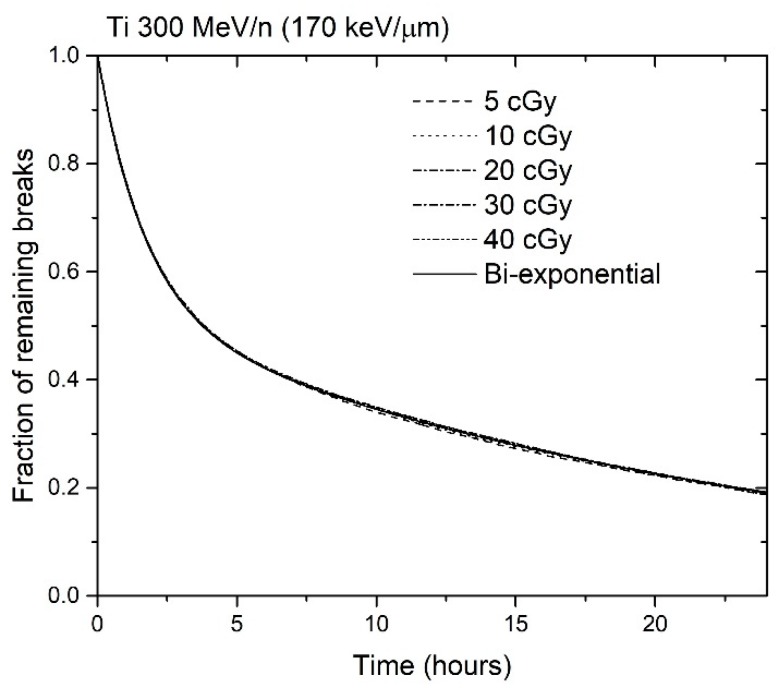
Fraction of remaining breaks as a function of time, after 5, 10, 20, 30, or 40 cGy Ti 300 MeV/n (170 keV/µm). A theoretical bi-exponential curve is also shown. The results were obtained using 10,000 simulation histories for each dose.

An example of repair kinetics curve obtained in the RITCARD simulation is shown in Figure 5.

**Figure 6:**
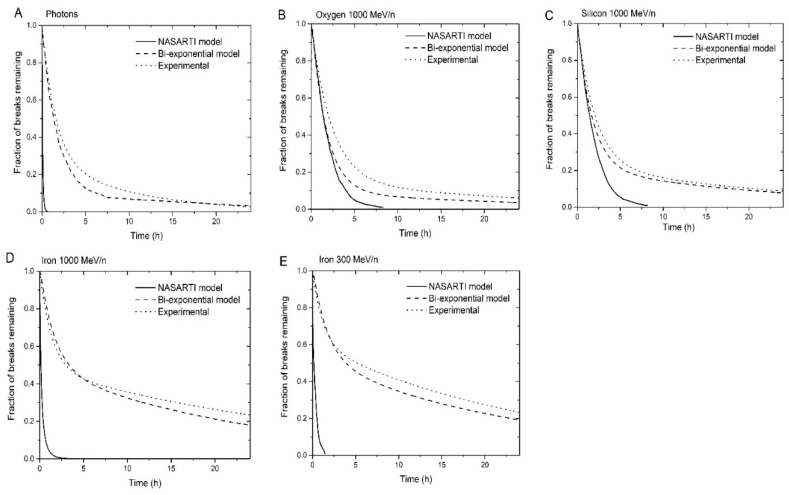
Repair kinetics obtained using the new restitution algorithm with a dose threshold set at 500 eV, and the results obtained from the former NASARTI algorithm and the experimental fit described in reference [9]: Photons (**A**), Oxygen 1000 MeV/n (**B**), Silicon 1000 MeV/n (**C**), Iron 1000 MeV/n (**D**), and Iron 300 MeV/n (**E**).

The results shown in Figure 5 illustrate that the breaks are repaired with the same kinetics regardless of dose, as the curves are nearly indistinguishable from each other and from the theoretical bi-exponential curve. In the dose range that we analyzed, the number of breaks is roughly linearly proportional to the dose, so that the initial number of breaks varies from 1.67 at 0.05 Gy to 13.15 at 0.4 Gy. However, when the repair kinetics curves are normalized to the initial number of breaks as shown in Figure 5 and Figure 6, they essentially follow the theoretical bi-exponential curve. This was not the case using the former algorithm (result not shown).

Repair kinetics curves for experiments reported in reference [9] are shown in Figure 6.

In Figure 6, we see that the number of unrepaired breaks at 24 hours are dependent on the LET. In general, higher LET leads to more complex breaks, which take longer to repair. Therefore, the number of unrepaired breaks 24 hours after simulated irradiation is higher for high-LET ions. With the previous algorithm, the breaks were mostly all repaired after 24 hours, regardless of the LET.

### 3.2. Fractions of Breaks Remaining at 3 h

**Figure 7:**
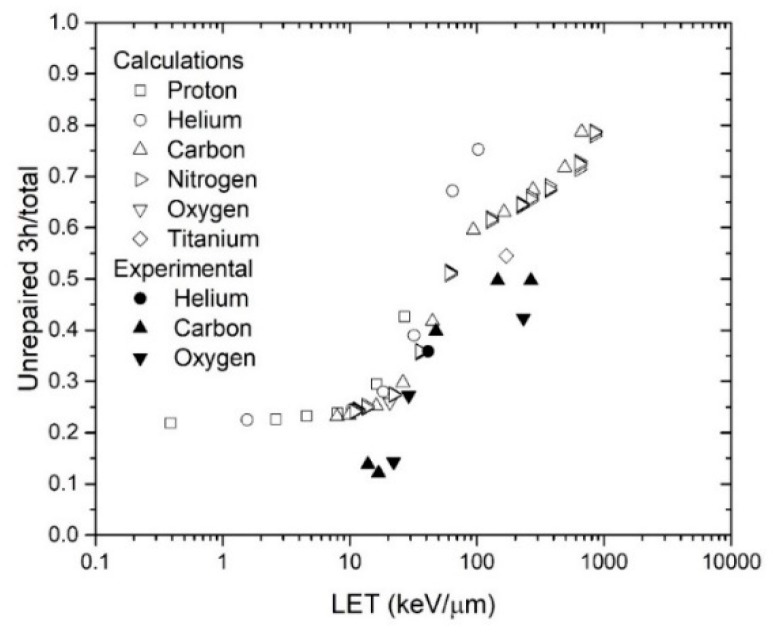
Fraction of remaining breaks 3 hours after irradiation with H, He, C, O, or Ti as a function of the LET, as calculated by RITCARD. Experimental results from reference [10].

The fraction of breaks remaining 3 hours after simulated irradiation is also a function of the LET. Since some experimental results are available [10], we also evaluated this quantity for various ions of different energies, covering a wide range of LET. The results are shown in Figure 7.

In general, the fraction of breaks remaining 3 hours after irradiation increases as a function of the LET. This is similar to data reported in reference [10].

### 3.3. Yields of Simple Exchanges

**Figure 8:**
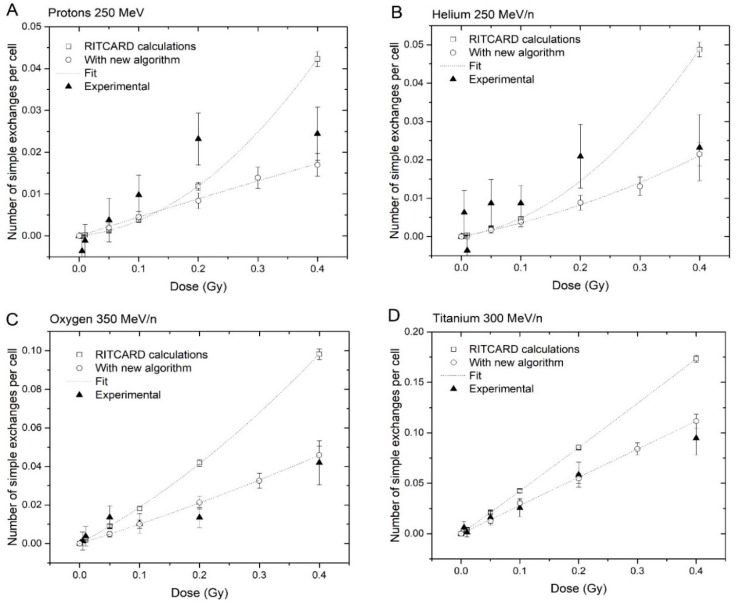
Comparison of RITCARD results obtained using the old and the new restitution algorithms, and experimental data for (**A**) protons 250 MeV, (**B**) helium 250 MeV/n, (**C**) oxygen 350 MeV/n, and (**D**) titanium 300 MeV/n.

The yields of simple exchanges were calculated using the original version of RITCARD, and compared with those obtained using the new version and experimental data. For clarity, it should be noted that all aberrations (simple and complex) involve improper rejoinings, which are considered complex breaks in the model. The simple breaks that are repaired leads to proper rejoinings and are not aberrations. Furthermore, a sequence of chromosome fragments may contain both proper and improper rejoinings. The results are shown in Figure 8. The experimental data used to compare in this work were obtained with 3-color FISH chromosome painting from human normal fibroblasts, and extrapolated to the whole genome using a modified version of the equation of Lucas et al. [18]. Furthermore, the background level of CA was subtracted by direct Monte-Carlo sampling. Detailed methods and original data were published in reference [6]. 

The data shown in Figure 8 were modeled using a linear quadratic model:

(9) $$CA=\alpha D+\beta D^{2}$$

In general, the simulation results using the new algorithm are in better agreement with the experimental data, especially at high LET. Additionally, the new algorithm produces a much smaller curvature of the linear-quadratic fit. Despite the overall improvement of the simulated results with the experimental data, there are still some discrepancies between the simulated and experimental results for H and He. However, we may notice that the yields for these ions are smaller than for O and Ti. Furthermore, the experimental error bars are quite large relative to the values, due to low yields. Other factors such as the background aberration rates and non-targeted effects can also be an issue here.

**Figure 9:**
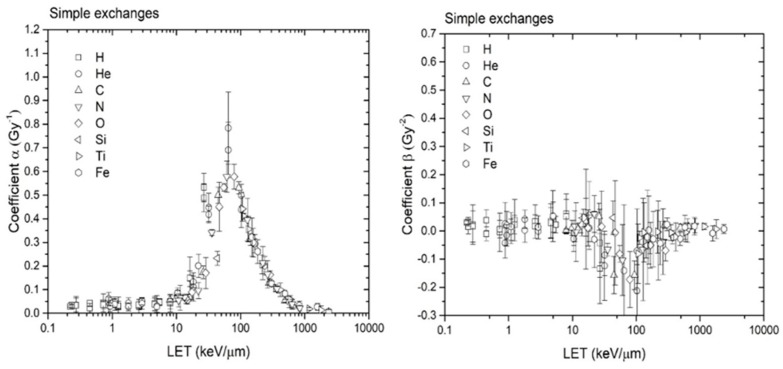
Coefficients *α* and *β* of the linear-quadratic model for simple exchanges plotted as a function of the LET.

Further CA dose–response calculations were performed with RITCARD for the ions H, He, C, N, O, Si, Ti, and Fe, at energies varying from 1 to 1000 MeV/n, covering a large range of LET values. The doses used are 0.05, 0.1, 0.2, 0.3 and 0.4 Gy for all cases. The *α* and *β* coefficients from the linear-quadratic fit for simple exchanges are shown as a function of the LET in Figure 9.

The α values calculated using the new version of RITCARD are similar to those calculated with the original version of RITCARD, which also located a peak at LET value near 100 keV/µm (results not shown). However, the coefficients *β* are markedly different from those calculated in the original version of RITCARD. Most *β* values calculated with the new version of RITCARD are between −0.1 and 0.1, and the calculated confidence intervals of the *β*s often includes 0. The overall trend is towards much smaller curvature or even a negative curvature. The experimental data (Figure 8, for example) show it is possible to obtain a negative value for the *β* coefficient for dose responses of simple exchanges. In contrast, the *β* coefficient given in reference [19] is, in general, significantly greater than 0; however, the investigators assessed much larger doses, up to 4 Gy. 

### 3.4. Influence of the Parameters of the Model

The model described in the present manuscript requires some parameters to be set. For the repair kinetics algorithm, only *τ_1_*, *τ_2_*, and the voxel dose threshold needs to be set. The parameters *A_1_* and *A_2_* are not set prior to calculations, but they are determined from the dose threshold and can be obtained a posteriori from the total number of breaks and the number of complex breaks. As *τ_1_* and *τ_2_* are considered intrinsic repair times for a type of cells, they are fixed prior to the simulations. The only free parameter for the repair kinetics algorithm is the voxel dose threshold; therefore, the new repair kinetics algorithm actually has fewer free parameters than has NASARTI. Having less parameters in the model usually indicates that the model is robust. Indeed, no change in the parameters is required for simulation of low and high LET radiations. We also remark that we took an average value for *τ_1_* and *τ_2_* for the calculations, whereas the experimental values varies from 1.18 to 2.33 hours for *τ_1_* and from 10.0 to 33.3 hours for *τ_2_*. Despite this variability in the parameters, the calculated repair kinetics curves are in relatively good agreement with the experimental results in all cases (Figure 6), so it appears that the results are not very sensitive to the precise values of *τ_1_* and *τ_2_*. The dose threshold may also be slightly too high. Reducing the threshold would likely increase the CA yields in all cases. That would improve the agreement between simulation and experimental results for low-LET ions (H and He) but may also worsen the agreement for high-LET (O and Ti).

The other parameters included in this model are the weight assigned to a given free end pair to repair, i.e., $\exp\left(-distance^{2}/\sigma^{2}\right)$, which is identical to the weight function used in NASARTI. Since this weighting factor concerns only free end pairs originating from complex breaks, this factor does not affect the repair kinetics; however, it does affect the repair of the free ends by favoring those in close proximity over distant ones and may, therefore, affect the yield of chromosome aberrations.

## 4. Conclusions

The repair of radiation-induced breaks is fundamental to understanding the formation of CAs. In this work, we have described the implementation of the mathematically exact bi-exponential repair kinetics model into the RITCARD model, thereby replacing the previous simplified repair kinetics approach carried over from the NASA Radiation Track Image (NASARTI) program. The new restitution algorithm solves the following problems with the previous algorithm: (1) the repair kinetics was a function of the initial number of DSB breaks; (2) with rare exceptions, all breaks in the system were repaired at 24 hours regardless of the LET; (3) despite having a function to simulate the decreasing numbers of DSB, the repair kinetics were not bi-exponential. Furthermore, implementing this new algorithm has simulated yields of simple exchanges that are closer to the yields from experimental results.

The bi-exponential repair kinetics model has been the subject of several debates for years, and no real consensus about the nature of the two types of damage was reached [1]. It was even considered an artefact by some authors. It was also shown that the repair process may be multi-component rather than dual, and that multi-exponential models improve data fitting significantly [20]. In the actual simulation model, adding exponential terms to the algorithm is straightforward. However, each exponential term increases the number of adjustable parameters, which would need to be determined in order for the model to be used. 

We apply the new model to a large number of ion types at various LETs and the resulting curvature of the dose−response curve for simple exchanges appears to be less than simulations obtained from the original model; however, this is difficult to verify at the doses considered (0.5 Gy and below) because the effect of the quadratic term is more apparent at higher doses. Furthermore, as in the previous calculations, the *α* component in the LQ model peaks at a LET value of about 100 keV/µm for simple exchanges.

This model may be able to predict the yield of chromosome damage that astronauts will incur from exposure to high LET space radiation, which is important because chromosomes aberrations have been validated as a marker of cancer risk. These simulations and future modelling efforts can also help us understand the mechanisms involved in the process of radiation-induced chromosome aberration formation. Most DNA repair is now known to involve complex multistep process involving many enzymes [21,22], whereas our model uses a simplified classification of breaks: simple and complex types. Obviously modeling such a complex enzyme system with atomic-scale DNA would represent a tremendous research effort. The model described here is relatively simple and easy to implement, yet it can predict the bi-exponential decay curves for several ions of different LET, and the fraction of breaks that remain unrepaired 3 h after irradiation.

## Figures and Tables

**Table 1:** Bi-exponential parameters.

Ion	a_1_	1/τ_1_	τ_1_	a_2_	1/τ_2_	τ_2_	a_1_/(a_1_ + a_2_)	a_2_/(a_1_ + a_2_)	a_1_τ_1_/(a_1_τ_1_ + a_2_τ_2_)	a_2_τ_2_/(a_1_τ_1_ + a_2_τ_2_)
Fe 300 MeV/n	46.0	0.85	1.18	71.1	0.04	25.0	0.39	0.61	0.030	0.970
Fe 1 GeV/n	62.8	0.72	1.39	57.0	0.03	33.3	0.52	0.48	0.044	0.956
Si 1 GeV/n	93.0	0.48	2.08	28.0	0.04	25.0	0.77	0.23	0.217	0.783
O 1 GeV/n	103.6	0.47	2.33	20.1	0.04	25.0	0.74	0.26	0.324	0.676
γ ray	90.4	0.65	1.54	36.8	0.10	10.0	0.71	0.29	0.274	0.726

## Appendix A

Let assume the simulation is done with type 1 breaks first. The time (typically 24 hours) is divided in bins *b_i_* of intervals of 1 s. If we start a simulations of *N_Histories_* with $N_{1}\left(0\right)$ breaks, the number of breaks is expected to follow the exponential distribution, N1(t)=N1(0)exp(−tτ1). Now let assume the simulation is done as in Algorithm 1. Each time a break is repaired, the time *t_repair_* is recorded, the index of the bin is found and the bin $b_{i_{1}}$ is incremented, i.e., $b_{i_{1}}=b_{i_{1}}+1$. At the end of the simulation, the bins are normalized. If we divide each bin by $N_{Histories}*sizebin$, the integral over all bins is expected to be 1. To get the predicted distribution the bins are multiplied by the area $N_{1}\left(0\right)\tau_{1}$, so that the overall multiplicative factor for the bins is

(A1) $$N_{1}\left(0\right)\tau_{1}/(N_{Histories}*sizebin),$$

and

(A2) $$b_{i_{1}}^{final}=b_{i_{1}}N_{1}\left(0\right)\tau_{1}/(N_{Histories}*sizebin)$$

Now let assume we do the simulation with a mixture of breaks of types 1 and 2. If we would use algorithm 2 using $A_{1}=N_{1}\left(0\right)$, $A_{2}=N_{2}\left(0\right)$ and *A = A_1_ + A_2_*, the number of elements in the bin *b_i_* would be given by the sum of the contributions of breaks 1 and 2, weighted by their fractions. That is,

(A3) $$b_{i}=\frac{N_{1}\left(0\right)}{N_{1}\left(0\right)+N_{2}\left(0\right)}b_{i_{1}}+\frac{N_{2}\left(0\right)}{N_{1}\left(0\right)+N_{2}\left(0\right)}b_{i_{2}},$$

The multiplicative factor in this case is

(A4) $$(N_{1}\left(0\right)\tau_{1}+N_{2}\left(0\right)\tau_{2})/(N_{Histories}*sizebin).$$

Therefore,

(A5) $$b_{i}^{final}=\left[\frac{N_{1}\left(0\right)}{N_{1}\left(0\right)+N_{2}\left(0\right)}b_{i_{1}}+\frac{N_{2}\left(0\right)}{N_{1}\left(0\right)+N_{2}\left(0\right)}b_{i_{2}}\right](N_{1}\left(0\right)\tau_{1}+N_{2}\left(0\right)\tau_{2})/(N_{Histories}*sizebin),$$

so that the overall result is not equal to the expected sum of the bins from the contributions from breaks of types 1 and 2. However, if we use $A_{1}=N_{1}\left(0\right)\tau_{1}$, $A_{2}=N_{2}\left(0\right)\tau_{2}$ as a weighting factor in the algorithm, the elements in the bin *b_i_* are

(A6) $$b_{i}=\frac{N_{1}\left(0\right)\tau_{1}}{N_{1}\left(0\right)\tau_{1}+N_{2}\left(0\right)\tau_{2}}b_{i_{1}}+\frac{N_{2}\left(0\right)\tau_{2}}{N_{1}\left(0\right)\tau_{1}+N_{2}\left(0\right)\tau_{2}}b_{i_{2}},$$

Applying the same overall multiplicative factor, we now can see that the terms

(A7a) $$b_{i}^{final}=\left[\frac{N_{1}\left(0\right)\tau_{1}}{N_{1}\left(0\right)\tau_{1}+N_{2}\left(0\right)\tau_{2}}b_{i_{1}}+\frac{N_{2}\left(0\right)\tau_{2}}{N_{1}\left(0\right)\tau_{1}+N_{2}\left(0\right)\tau_{2}}b_{i_{2}}\right]\left[\frac{N_{1}\left(0\right)\tau_{1}+N_{2}\left(0\right)\tau_{2}}{N_{Histories}*sizebin}\right],$$

(A7b) $$b_{i}^{final}=\left[\frac{N_{1}\left(0\right)\tau_{1}}{N_{Histories}*sizebin}b_{i_{1}}+\frac{N_{2}\left(0\right)\tau_{2}}{N_{Histories}*sizebin}b_{i_{2}}\right]=b_{i_{1}}^{final}+b_{i_{2}}^{final}$$

In this case, the final bins are the sum of the contributions from breaks of types 1 and 2.
